# Genomic Selection for Dairy Cattle Behaviour Considering Novel Traits in a Changing Technical Production Environment

**DOI:** 10.3390/genes14101933

**Published:** 2023-10-13

**Authors:** Larissa Elisabeth Behren, Sven König, Katharina May

**Affiliations:** Institute of Animal Breeding and Genetics, Justus-Liebig-University of Gießen, 35390 Giessen, Germanykatharina.may@agrar.uni-giessen.de (K.M.)

**Keywords:** automatic milking system, biomarker, cortisol, genetic parameters, human–animal relationship, learning behaviour, maternal behaviour, milking speed, social behaviour, temperament

## Abstract

Cow behaviour is a major factor influencing dairy herd profitability and is an indicator of animal welfare and disease. Behaviour is a complex network of behavioural patterns in response to environmental and social stimuli and human handling. Advances in agricultural technology have led to changes in dairy cow husbandry systems worldwide. Increasing herd sizes, less time availability to take care of the animals and modern technology such as automatic milking systems (AMSs) imply limited human–cow interactions. On the other hand, cow behaviour responses to the technical environment (cow–AMS interactions) simultaneously improve production efficiency and welfare and contribute to simplified “cow handling” and reduced labour time. Automatic milking systems generate objective behaviour traits linked to workability, milkability and health, which can be implemented into genomic selection tools. However, there is insufficient understanding of the genetic mechanisms influencing cow learning and social behaviour, in turn affecting herd management, productivity and welfare. Moreover, physiological and molecular biomarkers such as heart rate, neurotransmitters and hormones might be useful indicators and predictors of cow behaviour. This review gives an overview of published behaviour studies in dairy cows in the context of genetics and genomics and discusses possibilities for breeding approaches to achieve desired behaviour in a technical production environment.

## 1. Introduction

Cattle behaviour consists of a complex network of behaviour patterns. Feeding, lying and activity behaviour are individual cow reactions in response to environmental and social challenges. Increasing advances in sensor and video camera technologies have facilitated the recording of feeding, activity, and reproductive behaviour (e.g., heat). These provide valuable indicators of health and animal welfare. Meanwhile, there is a wide range of technology available that is offered by different manufacturers (e.g., Delaval, Lely, Nedap Livestock Management) which is suitable for commercial and automatic milking systems (AMSs). Such technology allows for detailed recording of dairy cow behaviour in order to improve farm profitability. For example, the detection of rumination behaviour can help to minimise production losses and disease costs in dairy farms [1]. Feeding, activity, and reproductive behaviour are influenced by genetic and environmental factors, especially social dynamics within a herd.

Social behaviour in a herd reflects individual behavioural reactions in a group of two or more cattle (cow–cow interactions). Cow–cow interactions and social behaviour towards conspecifics are particularly important in AMSs with free cow traffic. For example, aggressive, dominant, or temperamental cows can block the path to the AMS for submissive herd contemporaries. This initiates herd restlessness and also affects farm profitability. The definition of temperament includes consistent behavioural and physiological responses due to differences observed between individuals in response to a stressor or an environmental challenge [2]. Hence, temperament can also be expressed as the animal’s behavioural responses to environmental or social stimuli [3] or as a response to human handling [4]. The behaviour response to human handling (cow–human interactions) plays a major role in dairy cattle farming due to its effects on cow health, labour time, and production efficiency [5]. Cow–human interactions include cow aggressiveness, fearfulness and temperament in response to human handling and during milking. The increasing rate of AMS installations in recent years worldwide [6] implies a significant reduction in direct physical cow–human contact. In contrast, cows’ behaviour response to technical systems (cow–AMS interactions) has become increasingly important. Behavioural reactions to the technical environment include, e.g., temperament, nervous and hesitant behaviour directed towards the milking robot, as well as learning behaviour, which influences cow traffic and management processes in an AMS. In this regard, “milking temperament”, defined as aggressive or docile behaviour during milking in the presence or absence of a person [7], is an especially important breeding trait. Consequently, milking temperament is included in overall breeding goals or selection indices of dairy cattle in several countries [8].

The technological enhancements in modern dairy farms are associated with increased herd sizes and a reduction in human time needed to take care of the animals. Thus, recording cow behaviour subjectively in the context of routine farm work is quite difficult for herd managers. There exist experimental behavioural studies in dairy cattle that monitored cow temperament in response to human handling or during milking [9,10]. However, new measurements for behaviour and temperament in the context of a technical environment raise several additional questions, especially with regard to trait associations among the different behaviour and temperament categories. For example, with regard to the new and challenging technical milking robot environment, dairy cattle farmers have raised the question of whether more active cows learn to cope more quickly with changing production systems. Consequently, it is imperative to study novel traits reflecting cow behaviour in response to the technical environment (e.g., learning and social behaviour in an AMS) from a genetic and genomic perspective. Knowledge about genetic components, e.g., for learning or social behaviour, can be used to enhance selection indices and selection strategies towards improved workability in technical systems. Moreover, novel physiological traits and objectively measurable biomarkers (e.g., neurotransmitters) might be excellent indicators to explain and predict cow behaviour patterns in response to environmental and social stimuli. Several published review articles addressed cattle behaviour from a genetics and genomics perspective [3,11,12]. However, recently published review papers did not focus on cattle behaviour traits and correlated indicator traits in the context of future changing technical production environments.

This review is an overview of dairy cattle behaviour studies, focusing on the genetic background of behaviour traits, which is especially important in technological housing conditions such as an AMS. Initially, we introduce the different behavioural components in dairy cattle with the potential for breeding and selection applications. The following chapters address phenotypic relationships among dairy cattle behaviour categories with milk production, reproduction, and health as well as the potential use of novel biomarkers as indicators for behaviour and animal welfare. Understanding the relationships and interplay among the different components of behaviour and among behaviour and economically important traits might contribute to a deeper understanding of the underlying biological processes and physiological mechanisms. The second part of this review paper summarises results from quantitative genetics and genomics studies of dairy cattle behaviour traits, including genetic parameter estimations, genome-wide associations (GWASs), transcriptomics, and epigenetics. Specific chapters are supplemented by studies conducted in beef cattle since there is a gap of knowledge in the dairy cattle sector specifically addressing biomarker experiments or additional behaviour components such as maternal behaviour. Links to humans, rodents, and other livestock species are also made regarding innovations in phenotyping, genomics, biology and physiology of behaviour. Finally, the possibilities of including (novel) behaviour traits in selection indices and genomic selection programs is discussed.

## 2. Components of Dairy Cattle Behaviour

**Figure 1:**
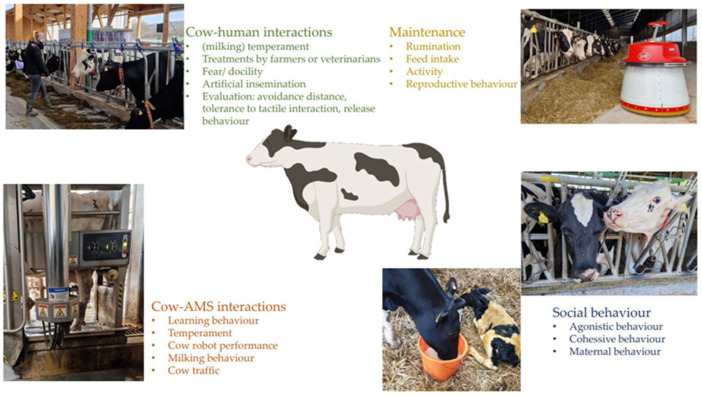
Behavioural components in dairy cattle. Dairy cow behaviour is composed of various complexes: maintenance, feeding, activity and reproductive behaviour, social behaviour, cow–human interactions, and cow–AMS interactions.

Dairy cattle behaviour in a technical environment is composed of various factors including their maintenance, feeding, activity, and reproductive behaviour, as well as their social behaviour towards humans, herd mates, or their offspring, and cow–AMS interactions (Figure 1).

### 2.1. Feeding, Activity and Reproductive Behaviour

The feeding behaviour of cattle is an important indicator to assess milk production and reproduction [13]. Feeding behaviour is induced by the circadian rhythm with several feeding periods throughout the day [14], and can be divided into three different phases: eating, ruminating and resting [15,16]. Eating is defined by chewing feed with the head in the feed bunk, or with the head away from the feed bunk, while ruminating means manipulating a cud with repetitive jaw movements that are not categorised as eating. Resting is defined as inactivity, and it ends with the initiation of either eating or rumination. Regrouping, stress, and disease can disrupt feeding behaviour [17]. In addition, learning ability, genetic disposition, quality and quantity of food and habits are factors affecting feeding behaviour [13]. Food quality and composition have been shown to affect behaviour, such as head butting and being butted at the feeding bunk [18]. Moreover, feeding behaviour is influenced by social dynamics in the herd, causing hesitant or reduced feed intake in low-ranked cows. Older and larger animals behave more dominantly while feeding than first-lactating and smaller cows [19]. Furthermore, dominant cows replace low- and middle-ranking herd members and spend more time at the feeding place, but their food intake is comparatively low [20]. The stocking density at the feeding bunk has a strong influence in this regard and affects feeding behaviour, especially dry matter intake, as well as the social behaviour of cows [21]. A high stocking density implies limitations of voluntary access to free space at the feeding fence, especially at times directly after feeding [22]. Hence, the time spent eating decreases with increasing stocking density [23], which is of practical relevance to improving both feed intake and behaviour. Commonly, feeding behaviour in dairy cattle (e.g., feed intake, rumination) is assessed by ear tags with electronic radio frequency identification systems [24], or by cow collars with transponders in AMS herds. Using sensor technology, further feeding behaviour traits can be recorded. This includes the number of visits to the feeding bunk or meals per day, duration and intake per meal, and feeding rate [15,25].

Activity and/or lying behaviour is a useful indicator of comfort behaviour and is influenced by housing, management, and environmental temperature [26]. From an economic perspective with the goal in mind to improve the health and welfare of dairy cows, it is imperative to optimise the time periods spent for feeding, lying, and activity [27,28]. Activity behaviour in dairy cows can be assessed by data loggers with an accelerometer located on the ear, neck, or leg [24,29]. Moreover, in the context of precision livestock farming, computer vision techniques can be used to visually observe behavioural changes in activity (e.g., reducing speed, dropping off the head while walking) in order to detect lameness and animal welfare restrictions at an early stage [30,31].

Due to the widespread use of artificial insemination, the accurate and timely detection of heat is the most important component of reproductive behaviour and is strongly related to farm economics. Heat behaviour is commonly measured by accelerometers, e.g., using the neck-mounted oestrus activity monitor *Heatime* [32]. Lovendahl and Chagunda et al. [33] showed strong correlations between activity from accelerometer data and heat and suggested that activity monitoring is helpful to manage herd fertility. According to Lucy [34], activity or standing behaviour alone is insufficient for oestrus detection, suggesting the need for evaluation of possible objectively measurable biomarkers as discussed in Section 4.

### 2.2. Behaviour Response to Human Handling: Cow–Human Interactions

Calm and docile cows are less stressed and less susceptible to injury than vicious cows, especially during restraint and handling [35]. Furthermore, fear unfavourably affects sexual and maternal behaviour in cattle [36]. In addition, the cow–human interaction can influence aspects of udder health [37], the success of first insemination [38] and the incidence of lameness [39]. We see a similar pattern in regards to cow health and labour required for treatments, e.g., claw disorder treatment is much more difficult with fearful cows. Dairy cows’ behaviour response to human handling is regarded to reflect the animal’s level of confidence or fear in humans [40]. The Welfare Quality^®^ Assessment protocol has been developed to evaluate the behavioural response of dairy cows toward humans, applying restrained or non-restrained methods or toward herd mates [41]. The recorded and generated behaviour data indicate the welfare status of the animals [41] and can be used to optimise animal handling and management decisions by humans [42]. For example, the avoidance distance to an unfamiliar person can be assessed at the feeding place [41] or in the barn [43,44], and give hints for the necessary size of barn constructions. In beef cattle, the traits “tolerance to tactile interaction” and “behaviour during release from restraint” are commonly used to monitor temperament [45], and were applied in dairy herds to describe cow–human interactions on a 5-point scale [11]. Behaviour traits reflecting the direct interactions between dairy cows and humans are listed in Table 1.

Behaviour response to human handling also includes characteristics related to milkability, i.e., milking temperament and milking speed (see Section 2.4 for a deeper discussion). Milking temperament is defined as aggressive or docile behaviour during milking in the presence or absence of a person [7]. Due to increasing numbers of dairy cows per herd worker [46] and increasing automatisation of routine processes as in milking robots, the direct contact between a cow and a herd employee is highly limited [47]. Nevertheless, cow behaviour is still affected by human actions in AMSs through the organisation of cow traffic [48] and through treatments by farmers or veterinarians [49]. Stress from routine processes can result in aggressiveness, nervousness, increased movement and reduced productivity [50,51]. In consequence, health, animal welfare and reproductive behaviour can be affected as well [52]. Hiendleder et al. [7] reported a decrease in longevity and decreased milk flow with increasing nervousness in Holstein Friesian (HF) dairy cow herds. Hence, it is important to reduce cow nervousness, fear, and aggression as a response to the actions or behaviour of humans or social partners in the herd also for economic reasons.

### 2.3. Social and Maternal Behaviour: Cow–Cow and Cow–Calf Interactions

With increasing herd size, understanding social behaviour in dairy cows (cow–cow interactions) contributes to improved farm management and selection strategies [53]. Cattle exhibit multiple social behaviours, such as licking, mounting, grooming, pushing, butting and chasing other herd mates, and fighting with them [54]. Social organisation in a herd directly (social interactions between individuals) and indirectly (changes in activity due to social interactions) influences milk production, reproduction and cow wellbeing [55]. Landaeta-Hernández et al. [56] showed that social stress influences progesterone levels and oestrus expression in cattle. Gibbons et al. [57] indicated that an animal with high social motivation and low aggression can better cope with group housing compared to less sociable animals. According to Kondo and Hurnik [58], the social hierarchy within a dairy herd is not only due to environmental components and animal experiences derived from related (agonistic) interactions but also by genetic predisposition. The Welfare Quality protocol^®^ is suitable for evaluating the expression of cow–cow interactions, with a strong focus on agonistic behaviour. Agonistic behaviour is defined as social behaviour related to fighting and includes aggressive as well as submissive behaviour patterns. Examples of agonistic behaviour are head butting, displacement, chasing and fighting. In contrast, cohesive behaviour is defined as behaviour promoting group cohesion and includes, e.g., social licking or horning without obvious agonistic intention [41].

Maternal behaviour (cow–calf interactions), i.e., the behaviour of the dam towards the offspring, is normally characterised by active and passive responses associated with the willingness to nurse and protect the offspring [59]. Jensen [60] investigated early postpartum behaviour in 38 multiparous Danish HF dairy cows and their calves. Recorded behaviours included: the duration and frequency of suckling, sniffing or licking the calf’s body or head, and social play. In intensive dairy production systems, maternal behaviour is of less importance since calves are separated from their dam within the first hours after birth. Nevertheless, the effects of maternal behaviour directly after birth might affect offspring production, health and social behaviour at later stages of life [61]. Interestingly, Weaver et al. [62] showed that maternal behaviour (e.g., licking, grooming) alters the offspring epigenome, and has an effect on stress responses of the offspring. Behaviour traits reflecting the cow–cow and cow–calf interaction are presented in Table 1.

**Table 1:** Behaviour traits describing cow–human, cow–cow, and cow–calf interactions in dairy cows.

Behaviour Trait	Interaction	Score	Reference
Avoidance distance at the feeding barrier	Cow–human	Distance [cm]	[41]
Avoidance distance in the barn	Cow–human	Distance [cm]	[43]
Tolerance to tactile interaction	Cow–human	1 to 5	[9]
Release behaviour after opening the feeding gate	Cow–human	1 to 5	[9]
Agonistic behaviour	Cow–cow	Number of aggressive behaviours per animal and time period	[41]
Cohesive behaviour	Cow–cow	Number of cohesive behaviours per animal and time period	[41]
Early postpartum behaviour of cow and calf	Cow–calf	Duration and frequency	[60]

### 2.4. Behaviour Response to the Technical Environment: Behaviour in (Automatic) Milking Systems and Learning Behaviour

In AMS, dairy cows meet new challenges that may necessitate the re-evaluation of existing traits or the integration of new traits into breeding indices. Dense phenotypic records from each AMS visit generate a longitudinal data structure for novel traits, which can be used to genetically improve the production efficiency in dairy farms [63]. Furthermore, AMSs provide the possibility to derive behaviour traits associated with cow robot performance, milking behaviour, and temperament [64]. Table 2 gives an overview of behaviour traits directly recorded in the AMSs. Several traits reflect the efficiency and functionality of a cow during milking. For example, one important trait is the ability to stay calm during preparation and attachment of the milking equipment. Hence, the milk production in kilogram produced per total box time, the attachment time, the milking frequency per day and the number of teat cup attachment failures, are indicators of the cow robot’s performance.

Specifically, milking behaviour can be described by the time entering and leaving the AMS (=box time), the milking duration of each AMS visit in minutes, the number of AMS visits per cow per day (=milking frequency), and the interval between milking sessions (=milking interval). Moreover, milk flow (average milk flow in kg min^−1^) and milking speed (milk yield per milk time) are important indicators for milking behaviour. Agitated cows may refuse milk ejection, implying a reduction of milking speed, prolonged milking duration and prolonged box time. Automatic milking systems enable cows to voluntarily regulate their own daily routine and milking rhythm. Some cows will adapt quickly to the advanced milking technique while others may need more time to get used to it. This also depends on a cow’s experience with AMSs and the ability to learn in novel (environmental) situations. In this regard, the open field or novel object test can be used to test a cow’s behavioural response to novelty or challenging situations. The open field test was also used to monitor cattle temperament since the temperament or emotional reactivity reflects an animal’s ability to cope with environmental changes [65].

In AMSs, the cow’s temperament is expressed by the number of unsuccessful milkings (=rejected/incomplete milkings), aggressive or docile behaviour during the milking process (=milking temperament), the time spent in the milking box before and after milking (=handling time), and the number of kick-offs during milking. Nervousness and kicking off the milking device prolong the handling time and decrease the cow’s efficiency [64]. Pedrosa et al. [63] introduced the trait ‘milking refusal’ as a further indicator for cow temperament and learning behaviour. Milking refusal means that a cow is not allowed to be milked because the expected milk yield is too low. A certain number of refusals per cow is considered to be normal and is caused by the animals’ adaptation to the AMS [63].

Cow flow or cow traffic is the most relevant factor contributing to alterations in feeding and movement behaviour in AMSs [66]. Disturbances in cow traffic are due to social behaviour and rank order within the herd or hesitant or fearful behaviour. A strong association exists between cows’ social rank and cow traffic [67]. High-social-rank cows spend more time chewing than feeding compared to low-social-rank cows, which are forced into the cow traffic routine. Consequently, access to feed concentrate stations affects cow behaviour and cow traffic in AMSs as well. Regarding cow traffic, Marino and Allen [68] showed that cows are unwilling to return to an area if they have an undesirable experience in this area (e.g., social aggression by group members). Moreover, it is theorised that cow traffic is mainly affected by learning behaviour, memories, and intelligence. Hence, the selection of improved learning behaviour of dairy cows in AMSs might favourably affect cow traffic, health, and production. However, currently available studies addressing learning behaviour in cattle are still limited [69,70,71].

## 3. The Relationship between Behaviour and Milk Production, Reproduction, and Health

Phenotypically, the relationships between behaviour, milk production, and reproduction were studied in different dairy cattle breeds. These studies, however, indicated inconsistent findings. Cziszter et al. [72] reported increased milk, fat, and protein yield in calm temperament cows compared to nervous cows in a dataset of 198 dual-purpose Simmental cattle. Moreover, nervous cows had significantly longer calving intervals, while the temperament had no effect on days open and the number of inseminations. Similarly, Mincu et al. [73] reported significantly higher milk production in calmer cows than in their nervous herd contemporaries in 94 Romanian Black Spotted lactating cows, while temperament had no effect on female fertility. Marcal-Pedroza et al. [74] estimated a significant negative correlation of −0.24 between the number of kicks during milking and milk yield in crossed Holstein-Gyr cows, implying higher milk yield in calmer cows. In contrast, in a study population including 12,028 Polish HF primiparous dairy cows, excitable and aggressive cows had higher daily and lactation yield compared to normal behaviour and calm cows [75]. In addition, milking speed increased with a smaller proportion of cows with calm temperament, and cows with more docile temperament tended to have shorter calving intervals and service periods [75]. Unfavourable phenotypic correlations between dairy cow behaviour traits indicating a cow’s fear of humans and economically important traits (e.g., milk yield) were reported by Breuer et al. [49]. Ebinghaus et al. [76] observed a tendency for faster average milk flow in cow herds characterised by a high percentage of cows with a short avoidance distance at the feeding fence in response to an unfamiliar person. In contrast to the studies mentioned above, Dutt et al. [35] identified no significant association between milking temperament and milk production and reproduction in a dataset of 81 Vrindavani cows.

Dairy cow behaviour can alter due to the disease status, which is summarised under the term ‘sickness behaviour’ [77]. Thus, cow behaviour might reflect the herd health status as well as the individual cow health status. Local pain induces changes in cow activity (e.g., change in lying behaviour to avoid pressure pain due to inflammation in the udder or claw), or changes in social behaviour (e.g., more aggressive/avoidance behaviour towards other cows in the herd or aggressive behaviour in response to human handling) [77]. Cows with mastitis spent less time lying down and ruminating and responded with a reduced water intake when the udder was swollen compared to the healthy control group [78,79]. Calderon and Cook [80] and Beer et al. [81] reported significantly longer lying times in lame compared to non-lame HF cows based on accelerometer data. Moreover, they identified an increased risk for ketosis in lame cows possibly due to significant differences in feeding behaviour (e.g., eating and rumination time) in lame compared to non-lame cows. Phenotypically, lameness was not associated with changes in social behaviour towards other cows from the same herd in HF cows [82]. Similarly, in AMSs, lame cows showed less feeding time and visited the robot less frequently compared to healthy cows [83,84]. Consequently, Garcia et al. [85] suggested including behaviour traits from AMSs (e.g., knocking off the milking device, voluntary entries) into artificial intelligence prediction models for early diagnosis of udder infections and claw diseases.

On the other hand, an animal may become sick due to individual behaviour or specific herd behaviour patterns (e.g., more aggressive cows in a herd due to human handling may explain an increase in diseased cows). Hence, increased disease susceptibility can be triggered by a certain behaviour. For example, in pasture-kept dairy cows, the type of grazing behaviour was related to the risk of helminth infections [86]. Moreover, specific behaviours can have a direct effect on physiological functions, and thus, indirectly alter disease susceptibility. More temperamental cattle displayed larger basal concentrations of stress hormones (e.g., glucocorticoids and catecholamines), which led to impaired immune cell functions [87]. Hence, farmers should generally select cows with a well-balanced temperament to achieve an improved overall herd health status. The associations among dairy cow behaviour, milk production, reproduction, health, and the involved neurophysiological pathways represent a complex network that is not yet sufficiently studied.

Furthermore, the gut microbial composition influences the host’s social behaviour and social interactions [88]. Due to the increasing importance of cattle microbiomes in the context of methane emissions and feed efficiency, associations between microbiome composition and host behaviour might be of future scientific importance.

## 4. Biomarkers as Indicators for Cattle Behaviour

**Figure 2:**
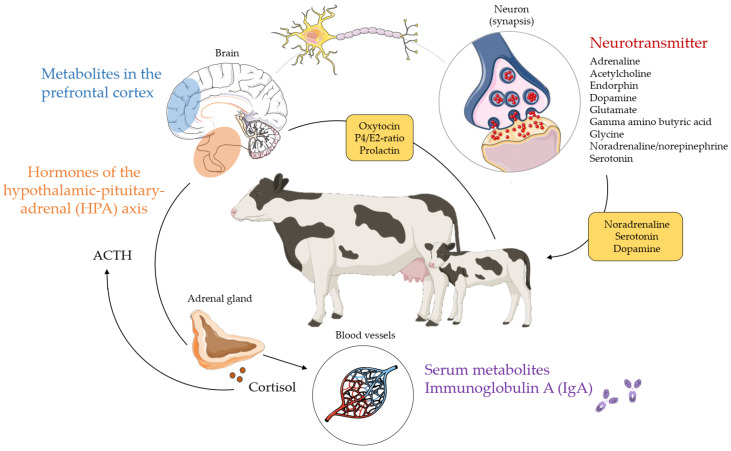
Molecular biomarkers known to be involved in dairy cow behaviour. The complex of behaviour molecular biomarkers includes hormones of the HPA axis, metabolites in the prefrontal cortex, neurotransmitters, serum metabolites and circulating IgA. The hormone ACTA stimulates cortisol production in the adrenal gland. Simultaneously, increased cortisol concentration in plasma negatively regulates ACTH production by the pituitary gland. The neurotransmitters are synthesised and released by neurons and act within synaptic gaps to transmit signals between neurons. Some neurotransmitters (e.g., dopamine, endorphin) act as both neurotransmitters and as hormones. Molecular biomarkers potentially involved in dairy cow maternal behaviour (verified in other animal species) are written in yellow boxes.

Cow behaviour underlies a complex network of neurophysiological reactions induced by fluctuations in chemical molecules such as hormones and neurotransmitters (Figure 2). Fluctuations in hormone levels and neurotransmitter concentrations stimulate psychological processes that induce physiological reactions (e.g., heart rate variability). Both physiological and molecular biomarkers have a genetic component as reviewed in Section 7, which represents the genetic influence on cow behaviour. To be able to follow the genetic and genomic mechanisms of physiological and molecular biomarkers in Section 7, an overview of studies addressing the biology and background of both categories will be given in this chapter.

### 4.1. Physiological Biomarkers

Psychological processes induced by social interactions and environmental changes activate several physiological mechanisms and can trigger stress reactions [89,90]. Depending on the individual’s behaviour to cope with stressful situations, the specific physiological stress response can vary among individuals. Heart rate and heart rate variability are physiological markers for the activation of the autonomic nervous system [90], and indicators for behaviour type and the status of animal wellbeing. Kovaćs et al. [91] presented a review of studies measuring heart rate and heart rate variability in dairy cattle. Heart rate was used as an indicator for behaviour in the case of cow–calf separation [92], and changes in heart rate during social interactions (e.g., licking) were also observed [93]. However, heart rate was not related to maternal ability [94]. In addition, heart rate is an indicator to study fearful behaviour (especially during handling [95,96]), and to compare restless behaviour between cows kept in automatic and conventional milking systems [97,98]. Moreover, increased heart rates and rectal temperatures indicate more excitable or temperamental cattle [52,99,100]. Changes in heart rate and body temperature may induce several pathophysiological effects and diseases as outlined in Section 3. Hence, physiological markers are suitable indicators for both trait categories of behaviour and health. Cattle heart rate can be measured utilising heart rate bells or by cardiac auscultation with a stethoscope. Rectal and intravaginal temperature are generally measured using a thermometer or intravaginal loggers. Jorquera-Chavez et al. [101] developed computer vision algorithms based on infrared and video techniques to assess heart rate, ear-base temperature, and respiration rate in dairy cows. The mean correlation coefficients between invasive methods (e.g., intravaginal loggers) and different computer-vision camera methods were in an acceptable range (mostly up to 0.6) for practical implementation [101]. However, since heart rate and rectal body temperature are often increased due to disease, molecular biomarkers might be more suitable indicators to infer changes in cow behaviour.

### 4.2. Molecular Biomarkers

The hypothalamic–pituitary–adrenal (HPA) axis and the sympathetic nervous system play a significant role in the body’s response to stressors and in psychological processes [87,102]. Hence, neurotransmitters and hormones of the HPA axis are indicators for behaviour and welfare [103,104]. Neurotransmitters include adrenaline, acetylcholine, endorphins, dopamine, glutamate, gamma amino butyric acid (GABA), glycine, noradrenaline (norepinephrine) and serotonin. Hopster et al. [105] showed lower plasma adrenaline and noradrenaline concentrations, and thus, less stressed behavioural and physiological responses in AMS cows compared to cows milked in a conventional tandem parlour. The role of dopamine and noradrenaline in aggression is well-known in mice and in other animal species (e.g., [106]). The dopamine and serotonin signalling systems are central to behavioural phenotypes such as temperament, as determined in Charolais cows [107]. Moreover, dopamine and serotonin are mainly involved in stereotypic cattle behaviour (e.g., repeated rolling of the tongue, and licking of stall equipment) [108]. Serotonin, the ‘happiness hormone’, modulates several cognitive and behavioural functions, e.g., activity, feeding, sleeping, social interactions, aggressiveness, learning and memory [109]. Increasing serotonin bioavailability can alter gene expressions of serotonin receptor genes and of immune-related genes as shown in pre-weaned dairy calves [110]. This finding suggests an important role of serotonin pathways in dairy cattle health. Breed differences seem to exist for dopamine secretion, with significantly higher levels in Simmental compared to Brahman and Nguni cattle [111]. The neurotransmitters noradrenaline, dopamine, and serotonin are involved in maternal behaviour in non-bovine species [112]. However, the role of neurotransmitters and their interplay with other hormones around the time of parturition in dairy cows is currently unknown.

Cooke et al. [113] demonstrated that excitable cattle exhibited higher plasma concentrations of the steroid hormone cortisol than calm cattle. The production of cortisol in the adrenal gland is stimulated by adrenocorticotropic hormone (ACTH), an important component of the HPA axis. Together with its precursor corticotropin-releasing hormone, ACTH is often produced in response to biological stress. For example, experiments on slaughter and transportation demonstrated that the elevation of ACTH concentration in plasma is a response to physiological stress in cattle [114,115]. The principal effects of ACTH are increased production and release of cortisol and androgens (e.g., testosterone) by the cortex and medulla of the adrenal gland. However, the detailed effects of psychological stress on ACTH and cortisol concentrations in plasma are not fully clarified. Boissy and La Neindre [116] demonstrated that social separation led to a significant increase in plasma cortisol concentrations, which was more pronounced in Aubrac than in HF heifers. In contrast, the separation of a cow from her calf had no effect on plasma cortisol in a study including eight HF multiparous dairy cows [92]. Instead, Coria-Avila et al. [59] showed that maternal behaviour (e.g., licking and nursing) is evoked by changes in the progesterone-estradiol (P4/E2) ratio, and by the oxytocin concentration. Besides progesterone and estradiol, the hormones oxytocin and prolactin are crucial to regulating maternal behaviour during the periparturient period [112]. Oxytocin is secreted from the posterior pituitary gland of the brain, while prolactin is produced by the anterior pituitary gland. While the role of oxytocin and prolactin in the formation of the maternal bond was clearly demonstrated in sheep and other animal species [112,117], it was not determined to be a useful biomarker for maternal behaviour in dairy cattle [94,112].

The effect of the hormone testosterone on fear and aggressive behaviour was reported in ruminants; however, results were inconsistent. Vandenheede and Bouissou [118] observed that testosterone-treated ewes were less fearful in behaviour tests, whereas Geburt et al. [94] associated higher levels of testosterone with higher docility in German Simmental and Charolais heifers. In dairy cows, testosterone treatment was used for oestrus detection but presents no common indicator to assess fear or aggressive behaviour.

Staley et al. [119] pointed out the role of Immunoglobulin A (IgA) as a biomarker for psychological stress. Serum, salivary or faecal IgA concentrations changed in response to social or production environment alterations (e.g., new social partner) in various pet and livestock species. Brand et al. [120] identified 54 prefrontal cortex and 51 serum metabolites having a high relevance in the classification of temperament types in HF × Charolais crosses. Specifically, differences in the abundance of metabolites related to C21 steroid metabolism between different cattle temperament types were identified. This may be a result of molecular pathway regulation involved in stress and fear response [120]. Additionally, in mouse models, metabolomics revealed brain metabolites, which are suitable indicators for stress resilience and depression [121,122]. In cattle, metabolomics can help to understand biological networks and the genetic architecture for economically relevant traits, aiming to use integrated “omics” analyses to improve practical breeding programs [123]. Hence, the detection of brain metabolites should be pursued further to integrate such data in “multi-omics” genomic-transcriptomic-metabolomic analyses, to improve dairy cow behaviour (especially in AMSs).

The secretion of neurotransmitters and hormones depends on genetics and gene expression, including epigenetic factors, as reviewed in Section 7. Vice versa, neurotransmitter and hormone concentrations can affect the expression of genes regulating the biosynthesis of other metabolites (e.g., [124]). In practice, biomarkers are not only suitable indicators for cow behaviour but can lead to substantial behavioural changes after supplementation via, e.g., feeding. For example, the neurotransmitter GABA can be supplemented orally to reduce aggressive behaviour in pigs and to increase dry matter intake in cows [125,126]. Hence, further research is needed to fully understand the role of neurotransmitters and hormones in cow behaviour, especially with regard to (learning) behaviour in AMSs. Moreover, AMSs allow a daily and detailed recording of parameters related to milk production and health. Researchers showed the potential of milk spectral data to predict pregnancy or disease (e.g., tuberculosis) based on milk composition, pregnancy and disease events combined with deep learning approaches (e.g., [127,128]. Similarly, using a training dataset of dairy cows where phenotypes and associations between molecular biomarkers and behaviour traits (as reviewed in Section 2) are well-known, it may be possible to derive behavioural characteristics from milk spectral data.

## 5. Genetic Parameter Estimates for Dairy Cattle Behaviour Traits and Genetic Correlations with Other Traits

### 5.1. Feeding, Activity, and Reproductive Behaviour

So far, little is known about the genetic variation of feeding behaviour and their genetic correlations with other traits in dairy cows, since most studies were conducted in beef cattle. Feeding behaviour traits in cattle include, e.g., time spent at the feeding bunk, duration of one feeding event, frequency of visits to the feed bunk, or dry matter intake per visit [129]. Heritabilities for such traits ranged from 0.11 to 0.61 in beef cattle [129,130,131]. Cavani et al. [25] estimated genetic parameters for nine feeding behaviour traits recorded in 1328 lactating HF cows with an automatic feed intake system over a period of ten years. The pedigree-based heritabilities for feeding behaviour traits ranged from 0.19 (number of meals per day) to 0.23 (feeding rate per visit). In this study, feeding behaviour traits were genetically closely correlated, i.e., breeding for more visits or meals per day implied less feeding time and lower feed intake per visit. However, the trait number of meals was genetically positively correlated with milk energy [25]. Lin et al. [132] estimated pedigree-based heritabilities in a range from 0.45 to 0.50 for feeding duration, number of visits, feeding rate and feed intake per visit in HF dairy heifers. Using sensor data, Yin et al. [133] estimated heritabilities of 0.02 for rumination, 0.20 for feeding, and 0.06 to 0.20 for different activity levels based on a multi-breed dataset. To our knowledge, there currently are no studies estimating heritabilities for heat and oestrus behaviour in dairy cows.

### 5.2. Behaviour Response to Human Handling

In HF, Dickson et al. [134] estimated a significant sire effect on cow reactions to human handling in the milking parlour, with heritabilities in a range from 0.45 to 0.53. Apart from this study, behavioural measures capturing aspects of cow–human relationships for breeding purposes in dairy cattle are strongly limited to the traits ‘milking temperament’ and ‘milking speed’. Estimated pedigree-based heritabilities for milking temperament ranged from 0.04–0.47 in dairy cattle milked in conventional milking systems [3,135]. For milking speed or milk flow, heritabilities ranged from 0.12 to 0.44 [136,137,138] when calculated using pedigrees or a genomic relationship matrix. Ebinghaus et al. [9,76] evaluated the suitability of different behaviour tests describing cow–human relationships for breeding (e.g., avoidance or distance toward an unfamiliar person, tolerance to tactile interaction). Moreover, the authors analysed phenotypic correlations between behaviour traits and milking temperament as well as milking speed. They concluded that a quality behaviour assessment could be a promising measure to improve breeding programs, but phenotypic correlations with milking temperament and milking speed were not significant [9,76].

### 5.3. Social and Maternal Behaviour

Kramer et al. [139] estimated genetic parameters for general temperament, aggression toward herd mates, and the herd rank order in Brown Swiss cattle. Estimated heritabilities were 0.38, 0.12, and 0.16, respectively [139]. Heritability estimates for dominance and aggression against other cows and for maternal behaviour in beef cattle and HF (crosses) ranged from 0.06 to 0.40 as reviewed by Haskell et al. [3]. The genetic relationship between maternal behaviour and reactivity to humans was close to zero, indicating that cows that lick their calves longer after calving are less reactive to human handling [140]. Nevard et al. [112] reported significant breed differences in maternal behaviour, with closer dam–calf relationships in beef compared to dairy cattle. This raises the question of whether differences in maternal behaviour exist among dairy cattle breeds.

### 5.4. Behaviour Traits Derived from (Automatic) Milking Systems

**Figure 3:**
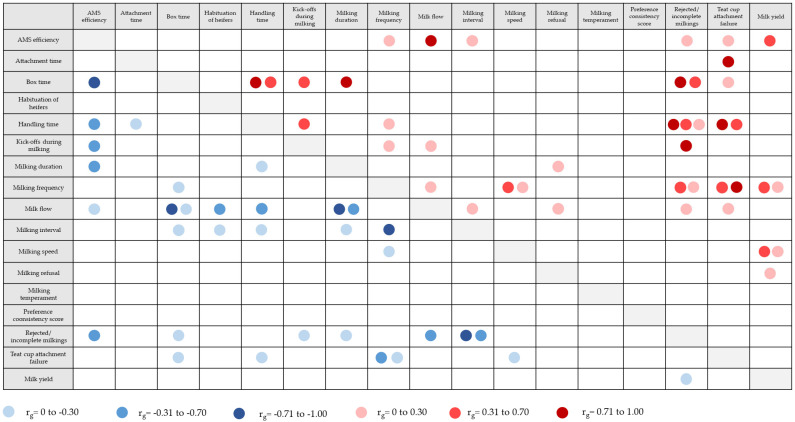
Genetic correlations among behaviour traits from Table 2 and between behaviour traits and milk yield. The correlations are based on references listed in Table 2. Circles indicate the range of genetic correlations. Positive genetic correlations are presented above the diagonal and negative genetic correlations are presented below the diagonal.

Data derived from automatic and technical systems such as AMSs imply a more accurate estimation of genetic parameters for milking behaviour traits because it provides a dense and longitudinal data structure considering each AMS visit [141]. Table 2 presents an overview of estimated heritabilities for behaviour traits recorded in AMSs (for trait explanations, see Section 2.4). Figure 3 gives an overview of genetic correlations among AMS behaviour traits, and between AMS behaviour traits and milk yield based on estimations from the references listed in Table 2. Several of the novel AMS traits that are closely correlated with milkability, milking temperament, and AMS efficiency showed pronounced genetic variation. Heritabilities for AMS efficiency and teat cup attachment failure were moderate and ranged from 0.22 to 0.56 (Table 2). Genetic correlations between AMS efficiency and temperament traits (e.g., handling time, and kick-offs during milking) were negative (−0.45 to −0.58). Thus, cows with appropriate behaviour keep calm during milking, implying higher milk production per total box time [64]. Piwcynski et al. [10] estimated a moderate heritability of 0.26 for the trait “time used for attaching the milking equipment”.

For AMS traits linked to milking behaviour, heritabilities showed greater variation and ranged from 0.02 to 0.52 (Table 2). The lowest heritabilities were found for the milking interval and milking frequency [64,138,141], indicating a stronger influence of environmental factors on the cows’ milking rhythm compared to the genetic component. Heritabilities for milking frequency (i.e., AMS visits per cow per day or frequency of voluntary entries in AMS) ranged from 0.02 to 0.51 (Table 2). Milking frequency is also related to robot capacity. A high frequency limits the available milking capacity per cow, and a low frequency increases labour time to organise cow traffic [64]. Genetic correlations between milking frequency and milk production traits were positive in a range from 0.14 to 0.57, indicating that cows visiting the AMS more frequently had greater genetic merit for milk production [138,142,143]. For milking speed and milk flow, estimated heritabilities ranged from 0.25 to 0.52 (Table 2). Santos et al. [138] estimated a negative genetic correlation of −0.88 between average milk flow and milking time during a visit to the AMS, which means that fast milking cows (i.e., cows with a good temperament) spent less time milking in the robot. For the trait “box time”, heritabilities were 0.27 [64] and 0.41 [144].

For traits strongly related to temperament in AMS (e.g., handling time, rejected milkings), heritabilities were quite small in a range from 0.01 to 0.15 (Table 2). AMS temperament traits were genetically favourably correlated with milkability and health indicator traits, suggesting that udder healthy cows had fewer incomplete milkings and a lower handling time than diseased cows [145,146]. The traits “kick-offs during milking” and “incomplete milkings” were moderately negatively correlated with milking speed, suggesting that cows with a higher milk flow behave more docile during milking [64]. However, the current available studies addressing the genetic background of cow behaviour traits in AMS strongly focused on milking behaviour and temperament [64], while neglecting behaviour traits associated with social herd dynamics, learning behaviour, or cow flow in AMSs. Vosman et al. [147] introduced the trait “habituation of heifers”, which reflects the time period a heifer needs to become familiar with the AMS. The heritability for this trait was 0.07 and showed a positive genetic correlation of 0.83 with milking interval.

**Table 2:** Behaviour traits are recorded by automatic milking systems with corresponding heritabilities (sorted alphabetically by trait).

Trait	Definition	Link to Behaviour	Heritability	Reference
AMS efficiency	Milk production in kg milk produced per total box time	Cow robot performance	0.29	[148]
			0.33	[144]
			0.40–0.50	[149]
			0.45–0.56	[63]
			0.23	[147]
			0.29	[150]
			0.22	[64]
Attachment time	Time used for attaching the milking equipment	Cow robot performance	0.26	[10]
			0.36	[146]
Box time	Time between entering and leaving the AMS	Milking behaviour	0.41	[144]
			0.06–0.33	[151]
			0.27	[64]
Habituation of heifers	Time period a heifer needs to become familiar with the AMS	Learning/habituation behaviour	0.07	[147]
Handling time	Time in AMS before and after milking	Temperament	0.05–0.15	[145]
			0.05	[64]
Kick-offs during milking	Number of knocking off the milking device	Temperament, udder health	0.06	[152]
			0.03	[138]
			0.06	[64]
Milking duration/ milking time	Milking duration of each AMS visit in minutes	Milking behaviour	0.39	[153]
			0.19	[138]
			0.22–0.28	[63]
			0.32	[10]
Milking frequency	AMS visits per cow per day	Cow robot performance, milking behaviour, learning behaviour	0.23	[154]
			0.02–0.07	[141]
			0.26	[144]
			0.16–0.27	[142]
			0.12–0.28	[151]
			0.02–0.08	[143]
			0.51	[10]
			0.05	[64]
Milk flow	Average milk flow in kg min^−1^	Milking behaviour, temperament	0.43–0.52	[63]
			0.25	[138]
			0.48	[64]
Milking interval	Interval between milking sessions	Milking behaviour, Temperament, social dominance	0.09–0.26	[141]
			0.17	[152]
			0.07	[138]
			0.08	[147]
			0.02	[64]
Milking speed	Milk yield per milk time	Milking behaviour	0.42	[154]
			0.43	[10]
			0.46	[150]
Milking refusal	The cow is not allowed to be milked because the expected milk yield is too low	Temperament, learning behaviour	0.02	[63]
Milking temperament	Aggressive or docile behaviour during milking	Temperament	0.14	[152]
Preference consistency score	Milking box preference consistency/frequency of access to each milking unit in a given time period	Learning/habituation behaviour	0.05–0.13	[151]
Rejected/incomplete milkings	Number of unsuccessful milkings	Temperament	0.02–0.06	[145]
			0.01–0.02	[64]
Teat cup attachment failure/ number of attachments per teat	Teat cup attachment failure	Cow robot performance	0.21–0.31	[145]
			0.06	[152]
			0.26	[146]
			0.002	[64]

## 6. Genomic Regions Associated with Dairy Cattle Behaviour

To date, around 230 marker associations (QTL/SNPs) are mapped for the trait category “behavioural” according to the cattle QTL database (CattleQTLdb, Release 50, 2023). The mapped QTLs are located on all autosomes and on the X chromosome. According to the CattleQTLdb, the term “behavioural” comprises temperament, flight speed, maternal behaviour, aggressive behaviour, duration of exploration/activity/inactivity during novel object or open field test, vocalisation/standing alert/walking/running after social separation, flight from the feeder, and calf sucking reflex. In total, 15 studies are listed in the CattleQTLdb, seven of which were conducted in dairy cattle. Further traits for dairy cow behaviour (e.g., milking speed) reviewed in this paper are partly related to other trait categories in the CattleQTLdb [155].

However, the literature search for QTLs and genomic regions associated directly with behaviour in dairy cows is generally less successful. This is not only due to the limitations of objectively recorded behaviour traits but also because some traits are only partly influenced by cow behaviour. For example, milking speed is commonly described as a behaviour trait derived from AMSs. However, milking speed is influenced by both trait components of cow behaviour and the physiology of the mammary gland. This makes it difficult to infer gene effects on behaviour and gene effects on health or physiology. Another example in this regard addresses feed efficiency. The most popular trait to predict feed efficiency is residual feed intake (RFI). Residual feed intake is defined as the difference between actual feed intake and predicted intake based on body size and production level [156]. Illustrating trait complexity, feed efficiency is influenced by feed conversion, behaviour (e.g., rumination, meals per day, feed intake per meal), physical activity, and other effects [156]. Thus, for traits such as milking speed or feed efficiency which are influenced by a complex network of biological processes and genes, it is difficult to specifically identify the genes and pathways regulating only the behavioural component.

### 6.1. Feeding, Activity and Reproductive Behaviour

Li et al. [157] conducted a GWAS for RFI in HF dairy cows and identified a region on BTA 25 including the genes *CARD11* and *EIF3B*. Yin et al. [133] identified 13 and 8 genes on BTA 11, 17, 23, 27, and 29 for rumination and feeding, respectively, assessed by ear tag sensors in a multi-breed population of dairy and dual-purpose cows. In this study, rumination was detected by repetitive ear movement due to chewing and regurgitation, while feeding was electronically recorded through masticatory movement [133]. One of the significant genes associated with feeding, *RPS6KB2*, was differentially expressed in Angus cattle selected for low and high RFI [158]. Lindholm-Perry et al. [159] detected an SNP-affecting feed efficiency and temperament in beef steers, indicating the possibility of simultaneous genetic improvements of both efficient feeding and desirable temperament.

For the trait “basic activity” (defined as ear movement resulting from walking, head shaking or other movements) Yin et al. [133] reported *PPM1E* on BTA 19 as a main candidate gene. However, for behaviour traits related to lying and activity, it is difficult to assess whether the behaviour is favourable or unfavourable. A high level of activity does not allow for any conclusion to be made about friendly or aggressive interactions with other cows or correlations with other behavioural components. The question arises whether genomic selection for increased or decreased lying and activity behaviour makes sense in the context of animal welfare and financial aspects. Aspects of behaviour trait importance in this regard might differ across species. For example, a GWAS in humans identified several genes involved in physical activity and sleep duration, where genomic heritabilities for both traits were moderate [160]. Studying the genomic background of activity in humans is crucial to reducing costs in healthcare systems since physical inactivity is strongly related to obesity, type 2 diabetes, and cardiovascular diseases. In species used for food production, behaviour traits are also associated with milk production and disease susceptibility. Friedrich et al. [104] applied a GWAS in an F2 HF × Charolais crossbreed population for activity, inactivity, and exploratory behaviour observed via novel-object and open-field tests. They identified 41 SNPs on 21 chromosomes and demonstrated that SNP genotypes associated with less exploratory behaviour and higher inactivity promote significantly higher milk yield [104].

Kommadath et al. [161] conducted gene expression studies in the brain tissue of 28 primiparous HF heifers to identify genes involved in oestrus behaviour. Oestrus behaviour was assessed by a heat score: a combination of different traits including, e.g., restlessness, mounting other cows, standing heat, and sniffing the vulva of another cow. The genes *AVP*, *MCHR1*, *POMC* and *OXT* were differentially expressed and associated with oestrus behaviour. Interestingly, these genes are involved in socio-sexual behaviour, anxiety, stress and feeding motivation as well [161]. Woelders et al. [162] showed that the expression of oestrus behaviour in dairy cows is centrally regulated by oestradiol-activated genes expressed in the anterior pituitary and in the brain. Imran et al. [163] associated the *CYP19A1* gene with oestrus behaviour in buffalos and suggested molecular markers located in this gene to improve oestrus behaviour in buffalos via genomic selection. *CYP19A1* was associated with abnormal parental behaviour, abnormal emotions, and decreased aggression in mice, as well as with fertility in HF cows [164]. Hence, *CYP19A1* seems to be involved in several biological processes related to reproductive behaviour, encouraging ongoing studies in dairy cattle in this regard. Results from a GWAS for feeding and activity behaviour traits in dairy cows are summarised in Table 3.

### 6.2. Behaviour Response to Human Handling

As reviewed in Section 5.2, genomic studies addressing the cow behaviour response to human handling, are mostly limited to the traits “milking temperament” and “milking speed”. For milking temperament and milking speed, genomic markers were detected on a large number of chromosomes (Table S1), indicating the polygenicity of complex behaviour traits. Hiendleder et al. [7] identified SNPs on BTA 5, 18, 29, and X/Y for the linear scored behaviour traits (recorded in nine classes from one to nine) milking temperament and milking speed. Abo-Ismail et al. [165] detected 406 SNPs on BTA 3, 4, 5, and 13 associated with milking temperament, and 601 SNPs on BTA 5, 6, 8, 9, 10, 19, 28, and 29 associated with milking speed. They found 1308 (overlapping) genes and 12 pathways regulating both traits via association and enrichment analyses based on data from the Bovine 50k BeadChip [165]. In a whole-genome scan considering HF bulls with phenotyped daughters, significant SNPs for milking temperament were detected on BTA 1, 10, 19, 24, 26, and 27, and on BTA 4, 13, 19, 22, 23, 26, and 29 for milking speed. The gene *SLC18A2* was associated with both traits [166]. Marete et al. [137] identified the most significant SNPs on BTA 7, 8, 10, 14, and 18 associated with milking speed in French HF cows. In addition, 11 QTL on BTA 7, 10, 11, 14, 18, 25, and 26 affected milking speed [137]. Chen et al. [167] detected 40 and 35 significantly associated SNPs with milking temperament and milking speed respectively in a population of ~4000 North American HF dairy cows (Table S1). Jakimowicz et al. [136] associated seven SNPs located on chromosome X (explaining 1.9% of the phenotypic variance) with milking temperament, and 24 SNPs with milking speed (explaining 5.8% of the phenotypic variance) (Table S1).

### 6.3. Social and Maternal Behaviour

Kramer et al. [135] conducted a study in Brown Swiss cattle and identified *TAC1* as a potential candidate gene on BTA 4 for temperament towards herd mates, and the *SLC9A9* gene on BTA 1 for rank order within the herd. According to the ensemble genome database [168], *TAC1* was associated with decreased anxiety-related response in mice. *SLC9A9* was associated with increased grooming behaviour, increased stereotypic behaviour, abnormal (social play) behaviour, and abnormal social recognition in mice. Thus, regarding social behaviour in dairy cattle, the mechanisms of *TAC1* and *SLC9A9* should be studied in more detail in ongoing research. In beef cattle and HF × beef crosses, genes affecting social interactions, temperament or response to social separation have been carried out more intensively (e.g., [169,170,171,172]). Some of the identified genes were related to neurotransmitter pathways (e.g., type 4 dopamine receptor gene [*DRD4*]) or to the solute carrier gene family (e.g., solute carrier family 9 member A9 [*SLC9A4*], solute carrier family 18 member A2 [*SLC18A2*]), which is involved in neurotransmitter transport and in (abnormal) behaviour [173].

Until now, genomic studies addressing maternal behaviour only have been conducted in beef cattle (e.g., [174]). According to the CattleQTLdb, three QTL and two SNPs on BTA 3, 6, 8, 26, and X were associated with maternal behaviour in beef cattle. The results from a GWAS for social behaviour traits in dairy cows are summarised in Table 3.

**Table 3:** Genomic regions from a GWAS for feeding behaviour, social behaviour, activity behaviour, cow–human interactions, and learning behaviour traits in dairy (crossed) cows (sorted by chromosome, trait name, and chromosome position).

BTA	Trait	Chromosome Position	Associated Gene/QTL ^1^	Breed	Reference
1	Rank order in the herd	126.5 Mbp	*SLC9A9*	Brown Swiss	[135]
	Standing alert in response to social separation	0.4–0.6 Mbp	BM6438	HF × Charolais crosses	[169]
	Vocalisation in response to social separation	95.7–95.9 Mbp	MBMS4044	HF × Charolais crosses	[169]
3	Refusals per day in AMS	73.5–73.9 Mbp	*NEGR1*, *PTGER3*	HF	[175]
4	General temperament	14.9 bp	*TAC1*	Brown Swiss	[135]
	Standing alert in response to social separation	51.3–86.3 Mbp	*MAF50-DIK026*	HF × Charolais crosses	[169]
	Vocalisation in response to social separation	51.3–86.3 Mbp	*MAF50-DIK026*	HF × Charolais crosses	[169]
6	Rank order in the herd	8.4 Mbp	*-*	Brown Swiss	[135]
	Walking in response to social separation	4.0–31.0 Mbp	*DIK5076-BM1329*	HF × Charolais crosses	[169]
7	Vocalisation in response to social separation	62.0–62.2 Mbp	*INRA053*	HF × Charolais crosses	[169]
8	Aggressiveness	58.5 Mbp	*-*	Brown Swiss	[135]
	Walking in response to social separation	51.9–52.0 Mbp	*CSSM047*	HF × Charolais crosses	[169]
	General temperament	101.5 bp	*AKAP2*, *TXN*, *TXNDC8*	Brown Swiss	[135]
9	Vocalisation in response to social separation	28.1–45.4 Mbp	BM2504-UWCA9	HF × Charolais crosses	[169]
10	Vocalisation in response to social separation	20.9–38.0 Mbp	BMS528-TGLA378	HF x Charolais crosses	[169]
	Walking in response to social separation	52.2–67.7 Mbp	BM888-CSRM60	HF × Charolais crosses	[169]
11	Rumination	55,229,674 bp	*-*	HF	[133]
	Standing alert in response to social separation	48.3–66.9 Mbp	*ILSTS100-IDVGA-3*	HF × Charolais crosses	[169]
12	Refusals per day in AMS	53.0–55.0 Mbp	*SPRY2*, *POU4F1*	HF	[175]
		84.3 Mbp	*MYO16*, *CARS2*, *IRS2*	HF	[175]
13	Not active	79,178,395 bp	*-*	Multi-breed	[133]
16	Standing alert in response to social separation	87.7–101.8 Mbp	HUJ625	HF × Charolais crosses	[169]
	Vocalisation in response to social separation	46.4–66.5 Mbp	ETH11-BM719	HF × Charolais crosses	[169]
	Walking in response to social separation	7.6–7.8 Mbp	BM121	HF × Charolais crosses	[169]
17	Rumination	68,187,177 bp	*-*	Multi-breed	[133]
18	Rank order in the herd	29.4 Mbp	*-*	Brown Swiss	[135]
	Refusals per day in AMS	25.4–26.2 Mbp	*COQ9*, *CNOT1*	HF	[175]
		55.7–55.9. Mbp	*SLC6A16*, *PTH2*, *ALDH16A1*	HF	[175]
		60.0–63.2 Mbp	*MBOAT7*, *CNOT3*	HF	[175]
	Vocalisation in response to social separation	0–15.8 cM	*IDVGA-31-ABS013*	HF × Charolais crosses	[169]
19	Rank order in the herd	14.1 Mbp	*ACACA*, *TADA2A*, *DUSP14*, *SYNRG*	Brown Swiss	[135]
	Refusals per day in AMS	50.5–50.6 Mbp	*TBCD*, *ACTG1*, *RAC3*	HF	[175]
	Standing alert in response to social separation	40.4–52.1 Mbp	CSSM065-ETH3	HF × Charolais crosses	[169]
	Vocalisation in response to social separation	40.4–52.1 Mbp	CSSM065-ETH3	HF × Charolais crosses	[169]
	Walking in response to social separation	25.1–40.4 Mbp	BMS2142-CSSM065	HF × Charolais crosses	[169]
20	Aggressiveness	65.7 Mbp	*ADCY*	Brown Swiss	[135]
	Flight from feeder	45.6–62.3 Mbp	DIK15-BM5004	HF × Charolais crosses	[169]
23	Feeding	19,834,215 bp	*SLC25A27*	Multi-breed	[133]
25	Flight from feeder	19.6–33.0 Mbp	BM737-INRA222	HF × Charolais crosses	[169]
	Vocalisation in response to social separation	19.6–33.0 Mbp	BM737-INRA222	HF × Charolais crosses	[169]
26	Vocalisation in response to social separation	1.6–19.6 Mbp	ABS12-HEL11	HF × Charolais crosses	[169]
27	Rumination	37,283,994 bp	*THAP1*, *RNF170*,	Multi-breed	[133]
28	Flight from feeder	6.2–6.4 Mbp	BP23	HF × Charolais crosses	[169]
29	Flight from feeder	29.4–51.1 Mbp	DIK094-MNB101	HF × Charolais crosses	[169]
	Rumination	46,014,507 bp	*RPS6KB2*, *PTPRCAP*, *CORO1B*, *GPR152*, *CaBP4*, *TMEM134*, *AIP*, *PITPNM1*	Multi-breed	[133]
		49,036,680 bp	*ENSBTAG00000000776*, *MRGPRG*	Multi-breed	[133]
	Vocalisation in response to social separation	17.9–24.6 Mbp	RM044-MNB-166	HF × Charolais crosses	[169]

^1^ Genes are written in italics and QTLs are written in non-italics; annotation tools and databases for gene annotation and QTL detection differ in different studies.

### 6.4. Behaviour Traits Derived from (Automatic) Milking Systems

Although traits reflecting cow suitability to AMS have been under consideration for selection for some years, GWASs to infer the genomic background of behaviour traits derived from AMSs are still limited. Studies addressing GWASs for the traits of milking speed and milking temperament were conducted in conventional milking systems (or without milking system information), and are summarised in Section 6.2. Schafberg et al. [175] applied a single-step GWAS using 50 K genotypes for the trait “refusals per day” considering a dataset of 2245 HF dairy cows kept in one German large-scale dairy farm with 27 AMS units. The trait “refusals per day” (=milking refusal, see Table 2) was defined as the difference between AMS visits and milkings since AMSs reject milking cows when they visit the AMS unit too frequently. Schafberg et al. [175] hypothesised that “refusals per day” are strongly affected by the ability to learn. Thus, they included the cow’s experience with AMSs as a fixed effect in the model for genome-wide associations. Significantly associated SNPs for “refusals per day” were identified on BTA 3, 12, 18, and 19 and were annotated to 17 potential candidate genes (Table 3) which are involved in neuronal development or in neurodegenerative diseases. The most interesting genes were the neuronal growth regulator gene 1 (*NEGR1*) on BTA 3, CCR4-NOT transcription complex subunit 1 (*CNOT1*) on BTA 18, and Rac family small GTPase 3 (*RAC3*) on BTA 19. *RAC3* was associated with abnormal learning behaviour and intellectual disability in rodent models (e.g., [176]). Genomic studies for learning behaviour are often based on rodent models since learning behaviour in livestock species is difficult and time-consuming to assess. This shows the need for new technological approaches (e.g., the development of artificial intelligence models based on camera and sensor data). Moreover, an objective measurement method and a definition for learning behaviour traits is a further challenge in this regard. Nevertheless, learning behaviour traits such as “refusal per day” are novel and important traits for genomic selection. Blocking or visiting the AMS without the right to milk indirectly affects cow traffic, other components of behaviour, and farm economics. Hence, more detailed genomic studies with a focus on (learning) behaviour traits related to the adaptation to the technical environment in dairy cows are needed.

## 7. Genetics and Genomics of Biomarkers as Indicators for Cattle Behaviour

The physiological biomarkers heart rate and respiration rate were commonly used as traits indicating heat stress in quantitative-genetic studies in dairy cows [177,178]. Al-Kanaan [177] estimated heritabilities of 0.05 for respiration rate and 0.07 for heart rate in HF dairy cows based on pedigree data. Similarly, the pedigree-based heritability for respiration rate was 0.04 in HF cows in a study by Luo et al. [178]. Physiological biomarkers such as heart rate and respiration rate are rather a symptom in response to several influential factors (e.g., heat stress, disease, social stress) than a breeding trait describing a specific physiological condition (e.g., fear). However, Shen et al. [179] combined an open-field test and heart rate variability to explore the activity of the autonomic nervous system in emotional control in Brahman and Yunling cattle. They found the *SORCS3* gene as a main candidate gene involved in emotional control [179]. Chen et al. [103] applied a GWAS for blood neurotransmitter and hormone concentrations in Brahman and Yunling cattle. They identified 20 associated loci and 18 candidate genes for ACTH, cortisol, dopamine, glutamate, and serotonin. The strongest signal was identified for the glutamate concentration in the *MCHR1* gene, which was associated with anxiety-like behaviour and feed intake in mice [180,181], as well as with oestrous behaviour in HF dairy cows [161]. Further important genes in the study by Chen et al. [103] were *SLC18A2* as a critical mediator of dopamine dynamics and *HTR1F*, a G protein-coupled receptor involved in the release of ACTH. The *SLC18A2* gene is part of dopaminergic and serotonergic synapse pathways and was also associated with temperament traits in Charolais cows [107]. Interestingly, *SLC18A2* was identified in a GWAS for milking temperament and milking speed in HF cows [166]. In mice, impulsivity and aggression were associated with variants within the tryptophan hydroxylase (*TPH2*) gene, a key enzyme in brain serotonin synthesis [182]. For the dopamine β-hydroxylase gene (*DBH*), gene polymorphisms were associated with hyperactivity in humans [183] and with aggressive behaviour in dogs [184]. The *DBH* gene encodes for the enzyme dopamine-ß-hydroxylase, which converts dopamine to norepinephrine (which is released in response to stressful situations). Lourenco-Jaramillo et al. [185] re-sequenced the *DBH* gene and identified significant haplotype differences between Brahman and HF cattle, which might be a result of differences in temperament between the two breeds. Similarly, Sifuentes-Rincón et al. [186] found differences in genetic polymorphisms in different dopamine receptor genes between *Bos taurus* and *Bos indicus* breeds, which might be due to breed differences in aggressiveness and temperament.

Dopamine, serotonin, and oxytocin pathways are mainly involved in the genetic mechanisms of mammalian maternal behaviour [187]. However, studies showing associations between genes related to these pathways and maternal behaviour in cattle do not exist. Gene expression studies in sheep showed that the prolactin receptor gene (*PRLR*) was differentially expressed between ewes with normal and those with abnormal maternal behaviour [188].

Different genes and pathways are involved in the secretion of the neurotransmitter glutamate. Interestingly, Moreno García et al. [189] suggested the glutamate metabotropic receptor 5 (*GRM5*) gene to be a “grazing gene”, involved in grazing personality behaviour, home range and movement tortuosity in Hereford cattle. They conducted sequence analyses to identify variants in *GRM5*, which is involved in glutamate and neurotransmitter activity [189]. The glutamate ionotropic receptor kainite type subunit 3 (*GRIK3*) gene was identified as a candidate gene for signatures of selection for agonistic behaviour in beef cattle [190]. Since glutamate is commonly known as a neurotransmitter involved in learning and memory, variations in glutamate receptor genes should be further investigated in dairy cows especially when aiming to improve learning and adaptation in technical environments with a less human–cow relationship.

For the hormone cortisol in the serum of beef and HF cattle, the pedigree-based heritability was 0.13 [191,192]. The only available GWAS for serum cortisol concentration in cattle was conducted in Brahman and Yunling cattle, identifying SNPs on BTA 8 and BTA 16 [179]. Genome-wide association studies in humans and in the rainbow trout showed that cortisol secretion as a response to psychological stress is polygenically determined, with a SNP-based heritability of 9% [193,194]. Until now, most studies addressing the genetics and genomics of physiological and molecular biomarkers were conducted in beef cattle suggesting the necessity to apply GWASs for neurotransmitter and hormone concentrations related to behaviour in dairy cows.

## 8. Inclusion of Behaviour Traits into Genomic Selection Indices

So far, dairy cattle behaviour traits are rarely taken into account in genetic selection programs worldwide. Milking temperament is already integrated into selection indices in Australia and in the Nordic countries (Denmark, Sweden, Finland). The relative economic weight for dairy cattle temperament is 8.72% and largest in Australia. It ranges from 1.06 to 1.33% for HF, Jersey, and Red Dairy Cattle in the total net merit indices of the Nordic countries [8,195]. In Norway, temperament is weighted with 0.42% in the total net merit index for Norwegian Red [8]. In Canada, France, the United Kingdom, New Zealand, and The Netherlands, a scoring system for temperament in dairy cattle exists. However, temperament is not included in selection indices. In Germany, national estimated breeding values for milking speed and milking temperament have been officially published since 2004, but are not considered in the overall breeding goal. However, milking speed is weighted with 20% in the ‘RZRobot’ in Germany, which has been published since 2014. The ‘RZRobot’ is an AMS selection index to improve the selection of cows suitable for the milking robot. Similar selection approaches are in progress in other countries, i.e., to improve milkability based on sensor data from AMSs and to consider milking behaviour traits [63].

In Germany, milking speed and milking temperament are a combination of subjectively recorded producer data and objective measurements for kg milk per minute milking. Similarly, in other countries, temperament is recorded by farmers or other persons using various scoring schemes (e.g., score from 1 to 3, from 1 to 5, or from 1 to 9, or using the definitions ‘easy’ versus ‘uneasy’ or ‘nervous’ to ‘calm’) [8]. The main problem arises from the fact that temperament and other behaviour traits are not objectively measurable, which complicates the availability of precise phenotyping for breeding value estimation. Thus, it is imperative to have precise phenotype data on behaviour traits derived from technical sensors and AMSs. Dos Santos et al. [196] used a novel objective method called REATEST^®^ to measure reactivity in Guzerat cattle during weighing. The test is based on an electronic device with an accelerometer, which measures the frequency, intensity and temporary variation of movements for 20 s [196]. Moreover, Yin et al. [133] showed that rumination, feeding, and activity data recorded by ear tag sensors are suitable to identify promising candidate genes involved in behavioural processes. Moreover, SNP-based heritabilities for sensor feeding and activity traits were moderate, with estimates up to 0.20 [133]. As previously discussed in Section 4.2, predicting dairy cow behaviour phenotypes based on routinely available milk spectral data might be a further possibility, which should be explored in detail in ongoing studies.

However, it is not only trait recording that represents a challenge for the implementation of behaviour traits in breeding. Defining the traits may also present as challenging, especially when aiming to improve novel learning behaviour traits, e.g., cow traffic in AMS. In most cases, behaviour traits do not follow a Gaussian distribution and are recorded in classes (e.g., nervous versus calm). Therefore, estimated heritabilities for the same behaviour traits vary across studies, which, inter alia, are due to the applied statistical model. Although the heritabilities for temperament traits derived from AMSs are close to zero (Table 2), favourable genetic correlations with highly heritable traits (e.g., milking speed) allow for genetic improvement of AMS efficiency via breeding. Chang et al. [8] suggested that a composite trait combining several temperament traits may be more appropriate than basing selection on estimated breeding values for a single trait. For some behavioural complexes, the question remains how to define the optimal behaviour pattern in a breeding context. For example, for traits such as milking box preference [151] as an indicator for learning and habituation behaviour, it is not completely clear if the cow’s preference for one AMS unit is beneficial in regard to improving cow traffic and social cow interactions. A behaviourally “more flexible” cow might be more efficient in large-scale farms with increased technical equipment and a large number of AMS units. Genetic correlations between behaviour traits derived from AMSs were estimated in numerous studies (Figure 3), however with inconsistent findings. Nevertheless, some traits showed pronounced favourable correlations useful for genetic evaluation, e.g., with a genetic correlation of 0.98 between kick-offs during milking and incomplete milkings [64]. Genetic correlations among behaviour traits from technical systems as summarised in this review can be used for the development of appropriate selection indices for milking robot herds.

As reviewed in Section 4, neurophysiological biomarkers are promising indicators for cow behaviour and might be helpful in improving genomic selection for desired behaviour. However, neurophysiological biomarkers are often difficult to measure, and recording is time-consuming. Additionally, biomarker phenotyping is too expensive and difficult to implement for a large number of animals, which is necessary to estimate accurate breeding values. Nevertheless, genomic studies revealed genes (e.g., *SLC18A2*) involved in neurophysiological pathways (e.g., serotonin signalling pathway) (see Section 7), which are very likely to cause phenotypic variability in behaviour. The renewed availability of sequence data allows for the study of genomic variation in such “core genes” and its association with cow behaviour. Recently, in humans, Rancelis et al. [197] suggested genomic prediction models for behaviour considering or specifically weighing “core genes”. According to the authors, GWASs often result in false positive signals. This justifies a higher weight of core genes, which are well-known to be involved in neurophysiological processes [197]. As tested for other complex functional traits in dairy cattle, it is possible to use training datasets with a limited number of phenotyped cows for hormone and neurotransmitters for ongoing GWASs to detect “core genes” involved in behavioural processes. Afterwards, the detected SNPs and variants can be added to bovine genotyping arrays as it was suggested and partially put into practice for other complex functional traits (e.g., endoparasite infections in Black Pied dual-purpose cows or methane emission using microbiome composition as a precise indicator) [198,199]. Moreover, training datasets for neurophysiological biomarkers allow us to estimate genetic correlations between promising biomarkers for behaviour and technically routinely available traits (e.g., rejected milkings, rumination). For instance, economically important traits and milking behaviour traits significantly correlated with dopamine and serotonin secretion. However, validated reference values are partially unknown for novel cow behaviour biomarkers, which makes it impossible to set a cut-off value for these objectively measurable traits. Nevertheless, using a smaller training dataset of deep-phenotyped cows may allow us to derive the best indicator traits for improved dairy cow behaviour and to predict dairy cow behaviour via, e.g., machine and deep learning tools as shown in neuroscience for other animal species [200].

Further research is urgently needed regarding breed differences and possible genotype-by-environment interactions (G × E) for behaviour traits in dairy cattle. Such knowledge is imperative when including behaviour traits in selection indices with optimal economic weights per breed, country, or production system. An example in this regard is the inclusion of temperament in HF, Jersey, and Red dairy cattle breeding goals in Nordic countries [8]. Although behaviour trait recording should focus on objective measurements in future breeding approaches, preliminary producer surveys are crucial to identify the most important traits related to farm economics and AMS efficiency. Moreover, the selection of cattle temperament types according to production system characteristics will improve animal welfare and overall productivity [36,201]. Van der Laak et al. [202] found no G × E for milking temperament between farms with grazing or indoor production systems in the Netherlands. Since grazing has a strong effect on feeding and social behaviour in cows [203], it might be interesting to study possible G × E for (milking) behaviour traits considering AMSs with and without grazing.

The environment also plays a role in epigenetic modifications, which affect behaviour as well. Vice versa, stress induced by social interactions or cow personality can lead to epigenetic modifications. Guan et al. [204] indicated that epigenetic regulation has an important role in learning and memory. In mice, epigenetic modifications implied changes in learning behaviour [205]. Future developments for the inclusion of epigenetic modifications in genomic selection strategies have been discussed for commercial breeding traits such as milk yield [206]. However, suitable methods to implement such approaches have not yet been developed due to the high costs of generating epigenetic data and the complexity of epigenetic modulations. Multi-omics studies should be carried out more intensively to identify key genes involved in dairy cow behaviour, which can be used to improve genomic prediction accuracy as already discussed for other complex traits in livestock breeding [207].

## 9. Conclusions

Dairy cow behaviour is due to complex physiologically and genetically determined processes. A cow’s behavioural response to the changing technological environment will become increasingly important in future because of digitalised and engineered farming systems. In this context, it is crucial to study cow–cow and cow–human interactions, as well as learning behaviour in AMSs more intensively. Maternal behaviour plays a subordinate role in dairy cows because of commonly separates the dam immediately from the calf. Nevertheless, it was indicated that maternal behaviour in dairy cows may affect production and health in the offspring via modulations of the epigenome. For the AMS traits “AMS efficiency”, “attachment time”, “milk flow” and “milking speed” (all of which are related to cow robot performance and milking behaviour), pronounced genetic variation exists. Heritabilities for temperament traits derived from AMSs are mostly close to zero, but from a breeding perspective, genetic correlations with economically important traits are mostly favourable. Regarding the future development of breeding strategies aiming to address desired cow behaviour and the challenges and costs of trait recording, we suggest a meta-analysis considering genetic correlations from all published studies. This review clearly indicates the complexity of behaviour traits and behaviour indicator traits, which is reflected by polygenicity, i.e., the large number of identified genomic regions. Genes related to the “solute carrier family” should be directly considered in genomic prediction models, since these genes are mainly involved in neurotransmitter transportation. Specifically, *SLC18A2* was shown to be associated with milking temperament, milking speed, general temperament and blood neurotransmitter concentrations in dairy and beef cattle. The use of molecular biomarkers as novel phenotypes to improve cow behaviour via breeding is limited, because of the challenge to implement large cow training datasets. Multi-omics analyses can be performed based on such training datasets to clearly identify genes and pathways involved in the physiological mechanisms that significantly contribute to specific behaviour patterns. From a practical point of view, farmers should invest in animal observation technology and select temperamentally well-balanced cows, also from a social-interaction perspective with herd contemporaries. Although the heritabilities are weak for some behaviour traits, we suggest the consideration of objectively measurable behaviour traits derived from AMSs (e.g., kick-offs during milking) in currently available selection indices.

## Data Availability

Not applicable.

## Supplementary Materials

The following supporting information can be downloaded at: https://www.mdpi.com/article/10.3390/genes14101933/s1, Table S1: Genomic regions from GWAS for the traits of milking speed and milking temperament in dairy (crossed) cattle.
