# Supplementary material for: Genomic Selection for Dairy Cattle Behaviour Considering Novel Traits in a Changing Technical Production Environment
Source: Genes (Basel). 2023 Oct 13;14(10):1933. doi: 10.3390/genes14101933 (PMC10606080; doi:10.3390/genes14101933)
Supplement: Supplementary file 1 [file genes-14-01933-s001.zip › genes-2649598-supplementary.pdf]

Table S1. Genomic regions from GWAS for the traits milking speed and milking temperament in dairy (crossed) cattle (sorted by chromosome, trait name and chromosome position). The traits were recorded in conventional milking systems, i.e., not in AMS, or information about milking system did not exist.

BTA	Trait	Chromosome position	Associated gene/QTL[1]	Breed	Reference
1	Milking speed	8,164,917 bp	*ADAMTS5*	HF	[167]
		128,420,783 bp	*RASA2*	HF	[166]
		134,451,951 bp	*LSS*	HF	[167]
		142,988,689 bp	*PDE9A*	HF	[136]
	Milking temperament	60,491,479 bp	*GAP43, LSAMP*	HF	[168]
		61,279,697 bp	-	HF	[168]
2	Milking speed	85,362,275 bp	*HECW2*	HF	[137]
	Milking temperament	41,433774 bp	*KCNJ3*	HF	[168]
3	Milking speed	26,331,630 bp	*CD101*	HF	[166]
		67,303,946 bp	*ZZZ3*	HF	[166]
		77,446,808 bp	*WLS*	HF	[166]
		120,122,176 bp	*OTOS*	HF	[137]
	Milking temperament	12,962,750 bp	*FCRL5, LOC100849046, LOC784007*	HF	[168]
		26,543,998 bp	*CD2, IGSF3*	HF	[168]
		61,388,404 bp	-	HF	[168]
		67,716,160 bp	*PIGK*	HF	[166]
4	Milking speed	18,630,065 bp	-	HF	[168]
		56,610,485 bp	-	HF	[168]
		70,201,858 bp	-	HF	[136]
	Milking temperament	40,499,442 bp	*NRCAM*	HF	[167]
5	Milking speed	28,975,642 bp	*LOC5122464, LOC112441543, HIGD1C, METTL74, TMPRSS12*	HF	[168]
		37,711,059 bp	-	HF	[136]
		75,361,846 bp	*CACNG2*	HF	[166]
		76,755,076 bp	*SYT10*	HF	[166]
		105,350,319 bp	*KCNA6*	HF	[136]
		105,474,132	*NDUFA9*	HF	[136]
		106,223,457 bp	*TIGAR*	HF	[137]
	Milking temperament	64,565,816 bp	*SCYL2*	HF	[166]
		65,301,359 bp	*ANO4*	HF	[166]
		68,461,765 bp	*CHST11*	HF	[166]
		68,586,846 bp	*CHST11*	HF	[166]

		70,338,965 bp	*RFX4*	HF	[166]
		71,778,071 bp	*SYN3, TIMP3*	HF	[166]
		71,810,896 bp	*SYN3*	HF	[166]
		82,513,431 bp	*MRPS35*	HF	[166]
		88,105,584 bp	*LOC520387*	HF	[166]
		95,149,822 bp	*RERG*	HF	[166]
		109,364,642 bp	*CACNA1C, IL17RA, CECR5, CECR1*	HF	[135]
		136 M	-	HF	[7]
6	Milking speed	1,623,443 bp	-	HF	[168]
		102,771,937 bp	*PPP2R2C*	HF	[136]
		103,186,091 bp	*ENSBTAG00000053771*	HF	[136]
		104,310,525 bp	-	HF	[136]
		104,350,026 bp	-	HF	[136]
		104,379,509 bp	-	HF	[136]
		110,478,351 bp	*LOC100298588*	HF	[166]
		110,828,142 bp	*CC2D2A, FBXL5 LOC100849050, BST1*	HF	[168]
		118,922,500 bp	*KIAA1329*	HF	[166]
	Milking temperament	32,808,304 bp	-	HF	[168]
		38,881,648 bp	-	HF	[168]
		99,732,094 bp	*PLAC8*	HF	[166]
7	Milking speed	45,196,738 bp	*POLR2E*	HF	[137]
8	Milking speed	59,424,073 bp	*VCP, FANCG, PIGO, STOML2, FAM214B, UNC13B*	HF	[168]
		70,535,700 bp	*PEBP4*	HF	[166]
		74,722,735 bp	*EPHX2, GULO HTD2, LOC781261, APTX*	HF	[168]
		74,730,092 bp	*DNAJA1, SMU1*	HF	[168]
		85,645,138 bp	*IPPK*	HF	[137]
	Milking temperament	89,513,243 bp	*NXNL2*	HF	[168]
		91,408,357 bp	*GRIN3A, PPP3R2*	HF	[168]
9	Milking speed	2,526,481 bp	-	HF	[168]
		16,815,501 bp	-	Brown Swiss	[135]
		73,175,040 bp	-	HF	[136]
		83,283,165 bp	*UTRN*	HF	[166]
		88,469,911 bp	*PPP1R14C*	HF	[137]
	Milking temperament	4,614,571 bp	-	HF	[168]

		29,540,351 bp	-	HF	[168]
		29,866,887 bp	*TBC1D32*	HF	[168]
		29,898,522 bp	-	HF	[168]
		62,471,145 bp	*RARS2, SLC35A1, CFAP206, C9Horf163*	HF	[168]
		101,306,064 bp	*PRR18, SFT2D1, MPC1, RPS6KA2*	HF	[168]
10	Milking speed	19,944,056 bp	*TGM1*	HF	[167]
		28,155,181 bp	*LOC788797, LOC788790, LOC788778, LOC504344, LOC788740, LOC521749, LOC516467*	HF	[168]
		29,028,329 bp	*RYR3*	HF	[166]
		53,949,424 bp	*MNS1*	HF	[137]
	Milking temperament	14,638,815 bp	*MAP2K5, SKOR1*	HF	[168]
		20,196,716 bp	*NEO1, HCN4, REC114*	HF	[168]
11	Milking speed	33,416,170 bp	*NRXN1*	HF	[168]
		55,348,499 bp	*CTNNA2*	HF	[168]
		76,490,540 bp	-	HF	[168]
		77,563,811 bp	-	HF	[168]
		81,666,608 bp	*FAM49A*	HF	[137]
		83,456,178 bp	-	HF	[168]
		86,130,607 bp	*LPIN1, NTSR2*	Brown Swiss	[135]
	Milking temperament	59,587,479	*LRRTM4*	HF	[168]
12	Milking speed	10,668,524 bp	*OLFM4*	Brown Swiss	[135]
		32,324,036 bp	*SGSG*	HF	[137]
	Milking temperament	80,284,325 bp	-	HF	[168]
		80,304,993 bp	-	HF	[168]
13	Milking speed	2,738,460 bp	*PAK5*	HF	[168]
	Milking temperament	32,497741 bp	*TMEM236*	HF	[166]
		60,045,226 bp	*MGC142355*	HF	[167]
14	Milking speed	8,264,685 bp	*ZFAT*	HF	[137]
		29,408,225 bp	*CYP7B1*	HF	[168]
		33,747,936 bp	*PRDM14, NCOA2*	HF	[168]
		33,891,204 bp	-	HF	[168]
		33,892,181 bp	-	HF	[168]
		65,573,091 bp	*YWHAZ*	Brown Swiss	[135]
	Milking temperament	49,856,629 bp	-	HF	[168]

		55,580,399 bp	-	Brown Swiss	[135]
16	Milking speed	2,876,485 bp	*TMCC2*	HF	[137]
		64,722,850 bp	*NCF2, ARPC5, RGL1, APOBEC4*	HF	[168]
	Milking temperament	18,812,423 bp	*KCTD3, USH2A*	HF	[168]
		18,815,839 bp	-	HF	[168]
17	Milking speed	47,753,340 bp	*PIWIL1*	HF	[166]
		74,598,498 bp	*DGCR2*	HF	[137]
	Milking temperament	56,150,431 bp	*HSPB8, SRRM4*	HF	[168]
18	Milking speed	32,867187 bp	*CDH11*	HF	[168]
		57,534,701 bp	*CEACAM18*	HF	[137]
	Milking temperament	58,270,112 bp	*BOSTAUV1R417, BOSTAUV1R418, BOSTAUV1R419 LOC112442216, LOC112442217, LOC101910094, LOC787057, LOC112442214*	HF	[168]
		105 cM	-	HF	[7]
19	Milking speed	7,250,802 bp	*ANKFN1*	HF	[136]
		7,300,658 bp	*ENSBTAG00000038823*	HF	[136]
		7,333,959 bp	-	HF	[136]
		7,717,717 bp	*TRIM25*	HF	[136]
		9,944,425 bp	*TRIM37*	HF	[136]
		10,691,042 bp	*VMP1*	HF	[136]
		19,814,327 bp	*TMEM97, IFT20*	HF	[136]
		32,348,116 bp	*HS3ST3A1*	HF	[166]
		41,647,314 bp	*GAS*	HF	[167]
		51,581,082 bp	*MAFG*	HF	[137]
		59,364,966 bp	-	HF	[136]
		59,437,500 bp	-	HF	[136]
		59,547,890 bp	*U6*	HF	[136]
		60,394,610 bp	-	HF	[168]
	Milking temperament	15,137,581 bp	*SLFN11*	HF	[166]
		37,837,691 bp	*CACNA1G*	HF	[167]
20	Milking speed	836,751 bp	*SLIT3*	HF	[168]
		6,586,964 bp	-	HF	[136]
		47,410,881 bp	-	HF	[137]
		62,244,291 bp	*CTNND2*	HF	[168]

		67,686024 bp	*ICE1*	HF	[168]
21	Milking speed	2,690,162 bp	*ATP10A*	HF	[137]
	Milking temperament	30,325,061 bp	*OTUD7A, ADAMTS7, TBC1D2B*	HF	[168]
22	Milking speed	6,287,984 bp	*OSBPL10*	HF	[168]
		59,712,580 bp	*GP9*	HF	[137]
	Milking temperament	46,425,659 bp	*CACNA1D*	HF	[167]
		59,471,127 bp	*EEFSEC, RUVBL1, SEC61A1*	HF	[168]
		59,619,868 bp	*KBTBD12, MGLL*	HF	[168]
23	Milking speed	28,460,633 bp	*PPP1R10, ATAT1, MRPS18B, ABCF1 PRR3*	HF	[168]
		28,463,360 bp	*GNL1, RPP21, LOC615278, LOC512672, BOLA-NC1*	HF	[168]
	Milking temperament	2,338,177 bp	-	HF	[168]
		13,897,540 bp	*BYSL*	HF	[167]
24	Milking speed	10,100,503 bp	*CDH19*	HF	[168]
		14,505,480 bp	-	HF	[168]
		20,051,012 bp	*HMCS*	HF	[167]
	Milking temperament	19,206,273 bp	-	HF	[168]
25	Milking speed	34,596,606 bp	*CCL24*	HF	[137]
		39,791,709 bp	*SDK1*	HF	[168]
26	Milking speed	34,495,734 bp	*SLC18A2*	HF	[167]
		44,539,739 bp	*LHPP*	HF	[137]
		46,250,006 bp	*C26H10orf90*	HF	[168]
	Milking temperament	34,495,734 bp	*SLC18A2*	HF	[167]
27	Milking speed	14,713,849 bp	*STOX2, ENPP6*	HF	[168]
		32,834,272 bp	*FGFR1*	HF	[167]
		40,306,840 bp	*RARB*	HF	[168]
	Milking temperament	35,413,178 bp	-	HF	[168]
		35,420,916 bp	-	HF	[168]
		35,737,738 bp	*ZMAT4*	HF	[168]
		35,737,851 bp	-	HF	[168]
		35,739,454 bp	-	HF	[168]
		39,668,668 bp	-	HF	[168]
		44,198,674 bp	*ZNF385D*	HF	[168]
28	Milking speed	24,678,582 bp	*HERC4*	HF	[137]
		28,953,159 bp	*MCU*	HF	[166]

29	Milking speed	45,629,483 bp	*CHKA*	HF	[136]
		49,779,067 bp	*TRPM5*	HF	[137]
	Milking temperament	8.3 – 15. Mbp	BMS764-BMC8012	HF	[7]
		23,068,761 bp	*LOC782544*	HF	[167]
		23,753,023 bp	*NELL1*	HF	[168]
		30,954,390 bp	*HNT*	HF	[167]
		36,737,805 bp	*LOC510943*	HF	[167]
		38,944,167 bp	*DPP3*	HF	[167]
		45,482,143 bp	*C11orf80*	HF	[166]
X	Milking speed	86,996,906 bp	-	HF	[136]
	Milking temperament	813,448 bp	-	HF	[136]
		863,652 bp	-	HF	[136]
		9 cM	-	HF	[136]
		62,929,712 bp	-	HF	[136]
		74,200,611 bp	*ENSBTAG00000026025*	HF	[136]
		74,239,322 bp	*TAF9B*	HF	[136]
		74,434,775 bp	*ATP7A*	HF	[136]
		131,311,943 bp	-	HF	[136]

[1]Genes are written in italic, QTLs are written in non-italic; Annotation tools and databases for gene annotation and QTL detection differ in different studies
